# Ethnic Variations in Severe Maternal Morbidity in the UK– A Case Control Study

**DOI:** 10.1371/journal.pone.0095086

**Published:** 2014-04-17

**Authors:** Manisha Nair, Jennifer J. Kurinczuk, Marian Knight

**Affiliations:** National Perinatal Epidemiology Unit, Nuffield Department of Population Health, University of Oxford, Oxford, United Kingdom; Indiana University, United States of America

## Abstract

**Background:**

Previous studies showed a higher risk of maternal morbidity amongst black and other minority ethnic (BME) groups, but were unable to investigate whether this excess risk was concentrated within specific BME groups in the UK. Our aim was to analyse the specific risks and to investigate reasons for any disparity.

**Methods:**

Unmatched case-control analysis using data from the United Kingdom Obstetric Surveillance System (UKOSS), February 2005-January 2013. Cases were 1,753 women who experienced severe morbidity during the peripartum period. Controls were 3,310 women who delivered immediately before the cases in the same hospital. Multivariable logistic regression modelling was used to adjust for known confounders and to understand their effects.

**Results:**

Compared with white European women, the odds of severe maternal morbidity were 83% higher among black African women (adjusted odds ratio (aOR) = 1.83; 95% Confidence Interval (CI) = 1.39–2.40), 80% higher among black Caribbean (aOR = 1.80; 95% CI = 1.14–2.82), 74% higher in Bangladeshi (aOR = 1.74; 95% CI = 1.05–2.88), 56% higher in other non-whites (non-Asian) (aOR = 1.56; 95% CI = 1.05–2.33) and 43% higher among Pakistani women (aOR = 1.43; 95% CI = 1.07–1.92). There was no evidence of substantial confounding. Anaemia in current pregnancy, previous pregnancy problems, inadequate utilisation of antenatal care, pre-existing medical conditions, parity>3, and being younger and older were independent risk factors but, the odds of severe maternal morbidity did not differ by socioeconomic status, between smokers and non-smokers or by BMI.

**Discussion:**

This national study demonstrates an increased risk of severe maternal morbidity among women of ethnic minority backgrounds which could not be explained by known risk factors for severe maternal morbidity.

## Background

Previous studies have shown a higher risk of maternal morbidity and mortality amongst non-white ethnic groups in the United Kingdom (UK) [1], [2]. However, these studies were unable to investigate whether this excess risk was concentrated within specific black and other minority ethnic groups (BME). The Office for National Statistics in the UK has projected the total population of BME groups to increase from 13% in 2006 to 28% in 2031 and as high as 44% by 2056 [3]. There has also been a continuous increase in the proportion of births to women born outside the UK since 1995 [4]. In 2012, the reported proportion was 25.9% which was more than double than that in 2000 [4]. Thus, it is important to understand whether there are specific risks of severe maternal morbidity in women belonging to BME groups to help devise effective prevention strategies.

Studies have also not been able to ascertain the causes that contribute to the increased risk of maternal morbidity among BME groups [1], [5]. Knight et al. postulated that the increased risk of severe maternal morbidity among non-whites compared to whites in the UK could be related to pre-existing medical conditions and factors related to care during pregnancy and child birth [1]. The aim of this study was to quantify the risks of maternal morbidity for individual ethnic groups in the UK and to further investigate reasons for any disparity including pre-existing medical conditions, past and current pregnancy problems, and healthcare utilization.

## Methods

Ethics statement: The London Multi-centre Research Ethics Committee approved the UK Obstetric Surveillance System (UKOSS) general methodology (04/MRE02/45) and the surveillance of individual near-miss maternal morbidities using UKOSS (04/MRE02/46, 04/MRE02/71, 04/MRE02/72, 04/MRE02/73, 04/MRE02/74, 04/MRE02/77, 07/H0718/54, 09/H0718/8, 10/H0717/20).

We conducted an unmatched case-control analysis using existing United Kingdom Obstetric Surveillance System (UKOSS) data collected between February 2005 and January 2013. The UKOSS methodology is described in detail elsewhere [6], [7]. Briefly, UKOSS was set up in 2005 to investigate uncommon disorders of pregnancy and ‘near-miss’ conditions [6]. Case notification cards are sent to all consultant-led obstetric units in the UK every month and the approach of ‘nil-reporting’ enables surveillance of different severe maternal morbidities. For every case reported, details are collected on a data collection form by the clinician responsible for managing the case. Rigorous follow-up of non-responders ensures a high level of data completeness [6].

**Figure 1:**
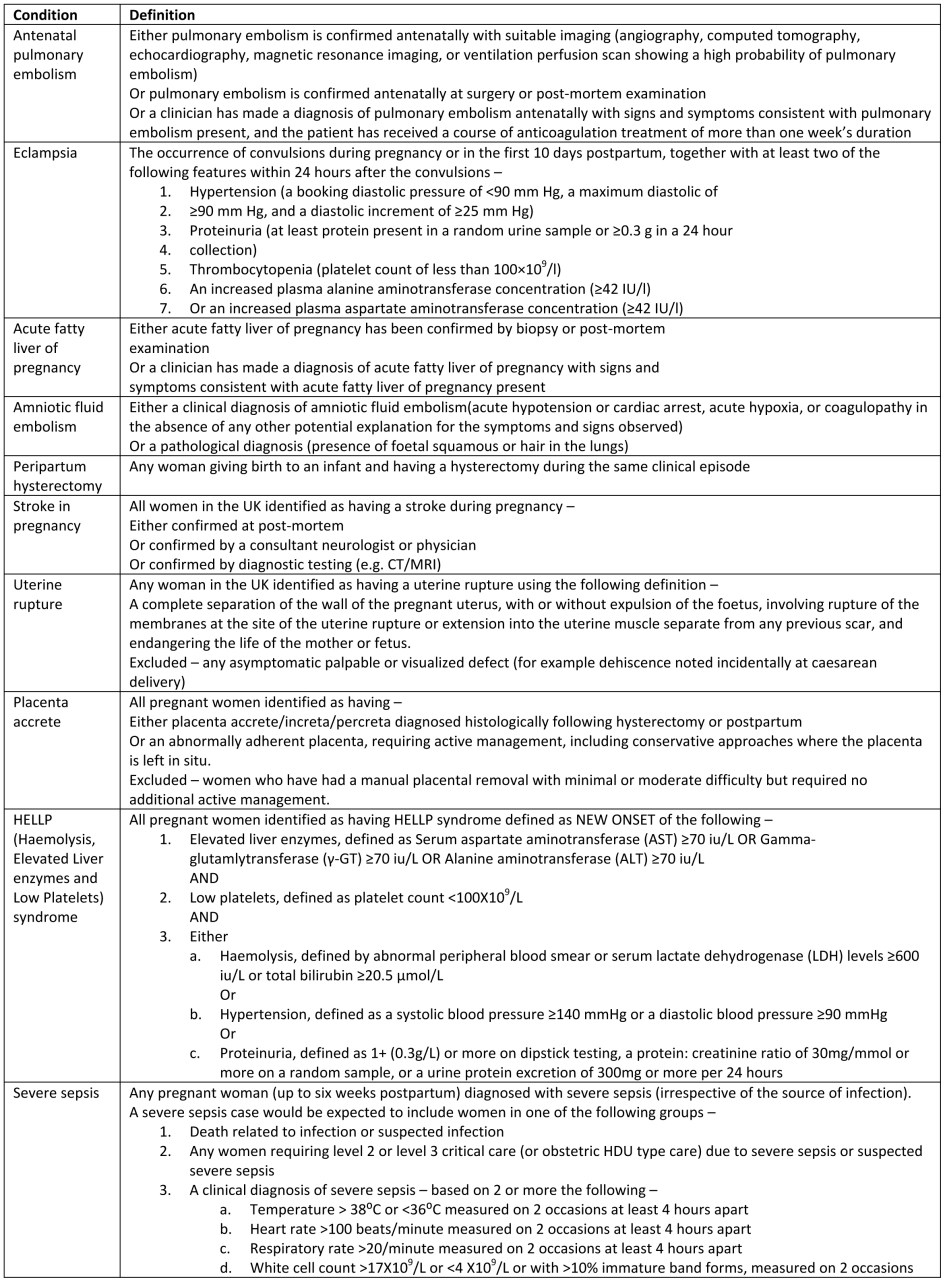
Box-1 – Definition of the conditions of severe maternal morbidity included in the analysis.

The cases included in this analysis were women who were reported to have one of the following ten conditions of severe maternal morbidity directly attributable to pregnancy causes: antenatal pulmonary embolism [8], eclampsia [9], acute fatty liver of pregnancy (AFLP) [10], amniotic fluid embolism (AFE) [7], peripartum hysterectomy [11], stroke in pregnancy [12], uterine rupture [13], placenta accreta [14], HELLP (Haemolysis, Elevated Liver enzymes and Low Platelets) syndrome and severe sepsis [15] (Table 1). Out of the ten conditions included, five had been included in an earlier study which showed a 50% increased risk of maternal morbidity among non-white ethnic groups compared to whites [1]. We undertook a preliminary analysis of the risk of morbidity among non-whites compared to whites in the new set of conditions (stroke in pregnancy, uterine rupture, placenta accreta, HELLP and sepsis) and since we found evidence of a similar level of excess risk among non-whites in this new set, the datasets of the ten conditions (five conditions included in the previous study and five new conditions) were merged and analysed together. The standard case definitions of these conditions as used in the UKOSS studies are given in box-1 (Figure 1). For all conditions, with the exception of AFLP, AFE and uterine rupture, the controls were women who delivered immediately before the cases in the same hospital [1]. The dataset did not have controls for AFLP and AFE cases, and we excluded the controls for uterine rupture because these were selected differently. In addition, we also included controls from a further study [16]. In total 1,753 cases and 3,310 controls were thus included.

**Table 1:** UKOSS studies from which data were included.

Sl. No.	Study	Cases/controls	Number of cases
1	Eclampsia	Cases and controls	214
2	Peripartum hysterectomy	Cases and controls	322
3	Acute Fatty Liver of Pregnancy/AFLP	Cases only^f^	51
4	Antenatal Pulmonary Embolism	Cases and controls	142
5	Obesity	*Controls only*	-
6	Stroke in Pregnancy	Cases and controls	37
7	Uterine Rupture	Cases only†	159
8	Placenta Accreta	Cases and controls	137
9	HELLP syndrome	Cases and controls	205
10	Severe Sepsis	Cases and controls	367
11	Amniotic Fluid Embolism/AFE	Cases only^f^	119

f Controls were not available;

† Controls were selected differently.

**Figure 2:**
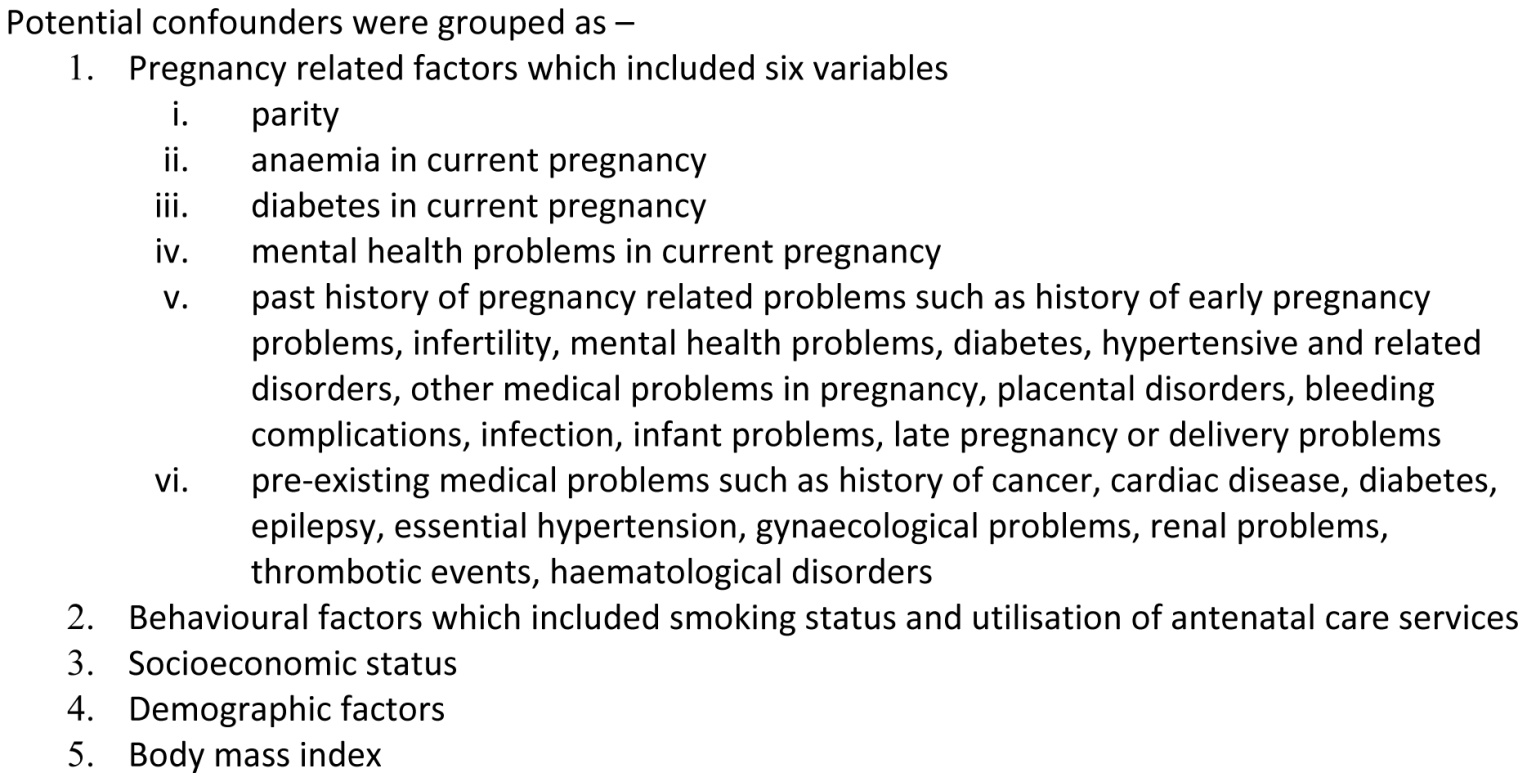
Box-2 – Potential confounders that were adjusted for in this study.

We categorised the ethnic groups based on self-reported ethnicity noted in medical records according to the UK national census classification [17]. Potential confounders (described in box-2 in Figure 2) were those that were adjusted for in previous studies [1], [5], [18]. Parity was included either as a continuous or ordinal variable with three categories, ‘primiparous’, ‘parity 1–3’ and ‘parity >3’ [18].

Similar to parity, BMI and maternal age were included either as continuous or categorical variables. Maternal age was categorised into <20 years, 20–34 years and ≥ 35 years [1], [18] and based on BMI at booking the women were grouped as underweight (BMI<18.5 kg/m^2^), normal/overweight (18.5 to 30 kg/m^2^) and obese (≥30 kg/m^2^) [19]. Socioeconomic status was based on the reported occupation of the women (or their partner if the woman was not in paid employment) and classified according to the National Statistics Socio-economic Classification system used in the UK [20].

Behavioural factors included were smoking and healthcare seeking. Women were classified as smokers or non-smokers. Information on concealed pregnancy, late booking and minimal antenatal care (ANC) were combined to create a binary variable ‘inadequate utilisation of ANC services’.

For the 1,753 cases and 3310 controls and considering a prevalence of 1.1% (exposure of the Bangladeshi ethnic minority group that constituted the lowest proportion), our analysis had 80% power to detect an odds ratio of 1.8 or greater associated with severe maternal morbidity at p <0.05 (two-tailed).

### Statistical Analysis

A univariable analysis was carried out to assess the association of ethnicity and other independent variables with the outcome. All variables found to be associated with the outcome at p-value <0.1 (two-tailed) and those identified in previous literature were used to build the multivariable logistic regression models.

Information on ethnicity was not available for about 1.8% of the sample. Knight et al. [1] included women with unknown ethnicity in the ‘white European’ group because the re-distributed proportions matched more accurately with the estimated ethnic profiles in the UK population census [21]. The same was done in this study. This method may not absolutely accurately classify women with missing information about ethnicity, but Moser et al. showed that the proportions of ethnic minority groups obtained by including maternities with missing ethnicity in white European group were in agreement with the profile of infants' ethnicity at birth [22]. Although for a majority of the independent variables the number of participants with missing data was <1% and their distribution did not differ significantly between the cases and controls and among the ethnic groups, for four variables – ‘socioeconomic status’, ‘BMI’, ‘smoking’ and ‘previous pregnancy problems’, the proportion of participants with missing information was >1%. On the basis of previous work [23], we assumed the data were not missing at random and coded ‘missing’ as a separate group for the categorical variables. In addition, a sensitivity analysis was performed for the four variables with >1% missing data by assuming extreme scenarios and accordingly redistributing the missing observations into the extreme groups; this had no significant effect on the findings.

Four multivariable logistic regression models were built to adjust for the identified confounders in a hierarchical fashion entering proximal factors first. In model–1 we adjusted for anaemia in current pregnancy, diabetes in current pregnancy, previous pregnancy problems, pre-existing medical problems and parity. In model–2 we added in smoking status and inadequate utilisation of ANC services. Model–3 included socioeconomic status in addition to those variables included in model–2 and model–4 adjusted for all variables including women's age and BMI. In addition to controlling for the known risk factors, this approach enabled understanding of their effects on the association between ethnicity and severe maternal morbidity. We tested for the most plausible interactions (between ethnicity and parity, ethnicity and smoking, ethnicity and socioeconomic status, socioeconomic status and smoking, and socioeconomic status and BMI) by adding interaction terms and using likelihood ratio testing (LR-test p<0.05); no significant interactions were identified.

**Figure 3:**
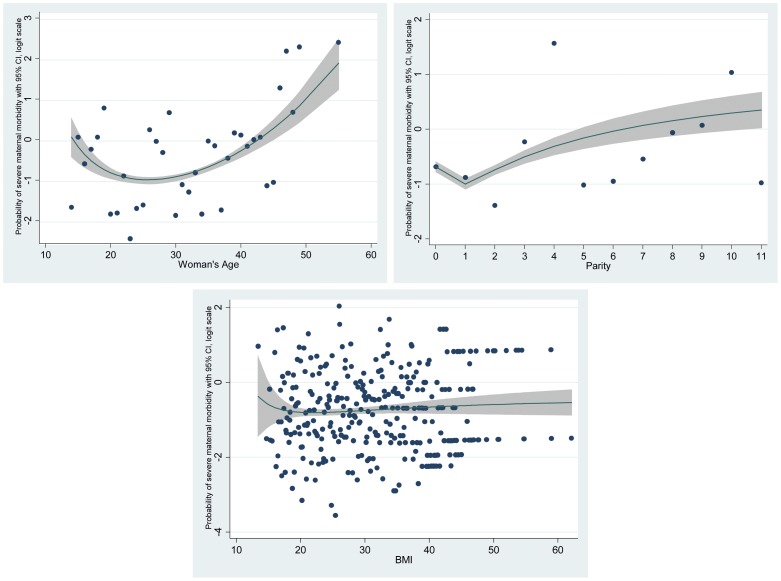
Patterns of association of women's age, parity and BMI with severe maternal morbidity.

We tested continuous variables for deviations from linearity by fitting functional polynomials in the univariable logistic regression models with multiple transformations of the continuous variable x^p^, where power (p) included −2, −1, −0.5, 1, 2, 3 and natural logarithmic transformation [24]. The best-fitting models for the continuous variables among 44 different combinations [24] suggested that there were non-linear associations between these variables and the outcome (Figure 3). We analysed both the best-fitting transformed continuous variables and the ordinal categorical forms in the multivariable logistic regression models and conducted subsequent LR-tests. The variables in one form or the other were not different in their confounding effects on the association between ethnicity and severe maternal morbidity and both forms of the variables indicated equally good fit (p-value of LR-test was <0.05). Thus, in the final model we incorporated these as categorical variables for the ease of interpretation of the odds ratios and the advantage of including and testing the missing data as a separate category. All analysis was done using Stata version 11 (StataCorp, College Station, TX) [25].

## Results

**Figure 4:**
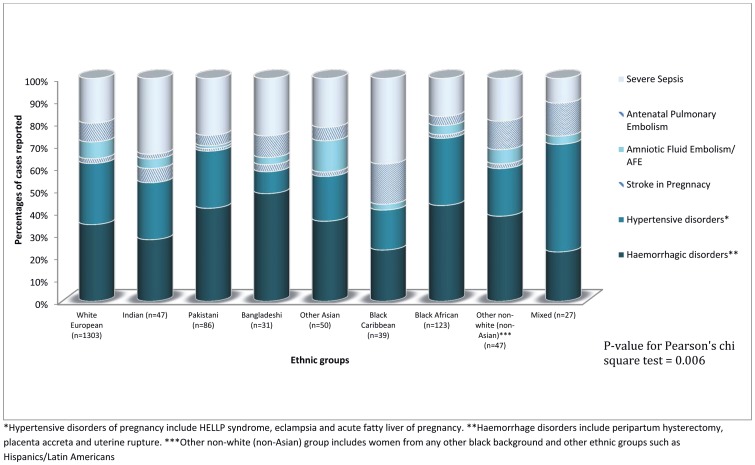
Contribution of different conditions of severe maternal morbidity among the different ethnic groups.

The distribution of the severe morbidity conditions differed significantly across the ethnic groups (Figure 4). While severe sepsis was most common among the black Caribbean and Indian women, peripartum hysterectomy, placenta accreta and uterine rupture, grouped as haemorrhagic disorders were most common among the Bangladeshi, Pakistani and black African groups. The ethnic groups differed in their characteristics (Table 2). Considering just the control women, a higher proportion of the black Caribbean women were in the extreme age groups at the time of delivery (either <20 years or ≥35 years). While a higher proportion of the Bangladeshi women were underweight, obesity was more prevalent in black Caribbean and black African women. A higher proportion of Indian women had a higher socioeconomic status (45% in managerial jobs), routine/manual class was higher in the Bangladeshi (30%) and other Asian (33%) groups, and unemployment was higher in the Bangladeshi (27%) black African (27%) and Caribbean groups (31%). In general, a lower proportion of women in the ethnic minority groups smoked compared to women with white European and mixed ethnic origins. Largely, the ethnic groups did not significantly differ with regards to the pregnancy related factors. A higher proportion of women with black African, other Asian and mixed ethnic backgrounds were diagnosed with diabetes during their current pregnancy. Proportionately more women of Pakistani, black African and black Caribbean origins had a parity of >3. About 6.5% of the black African, and 2% of Pakistani and black Caribbean women were grand multipara (≥5 pregnancies).

**Table 2:** Characteristics of control women by ethnic groups.

Characteristics	Ethnic groups – Number (%)											P-value^f^
	White European n = 2691	Indian n = 100	Pakistani n = 130	Bangladeshi n = 37	Other Asian n = 80	Black Caribbean n = 45	Black African n = 124		Other non-white (non-Asian) n = 61		Mixed n = 42	
**Demographic factors, behavioural factors and BMI**												
*Age*												
*<20 years*	138 (5.1)	1 (1.0)	1 (0.8)	2 (5.4)	1 (1.3)	5 (11.1)		2 (1.6)		1 (1.6)	5 (11.9)	0.006
*20 – 34 years*	1960 (72.8)	81 (81.0)	112 (86.2)	32 (86.5)	62 (77.5)	28 (62.2)		88 (71.0)		43 (70.5)	30 (71.4)	
*≥ 35 years*	573 (21.3)	18 (18.0)	17 (13.1)	3 (8.1)	17 (21.3)	12 (26.7)		34 (27.4)		17 (27.9)	7 (16.7)	
Missing	20 (0.7)	0 (0.0)	0 (0.0)	0 (0.0)	0 (0.0)	0 (0.0)		0 (0.0)		0 (0.0)	0 (0.0)	
***BMI*** †												
*<18.5*	72 (2.7)	3 (3.0)	2 (1.5)	4 (10.8)	5 (6.3)	0 (0.0)		4 (3.2)		2 (3.3)	0 (0.0)	<0.001
*18.5 – 30*	1922 (71.4)	84 (84.0)	102 (78.5)	25 (67.6)	69 (86.3)	23 (51.1)		75 (60.5)		43 (70.5)	30 (71.4)	
*≥ 30*	489 (18.2)	10 (10.0)	23 (17.7)	6 (16.2)	3 (3.8)	17 (37.8)		31 (25.0)		10 (16.4)	8 (19.1)	
*Missing*	208 (7.7)	3 (3.0)	3 (2.3)	2 (5.4)	3 (3.8)	5 (11.1)		14 (11.3)		6 (9.8)	4 (9.5)	
***Socioeconomic status (occupational classification (NS-SEC))***												
*Managerial*	701 (26.1)	45 (45.0)	25 (19.2)	5 (13.5)	17 (21.3)	8 (17.8)		20 (16.1)		14 (23.0)	9 (21.4)	<0.001
*Intermediate*	558 (20.7)	22 (22.0)	35 (26.9)	5 (13.5)	20 (25.0)	11 (24.4)		17 (13.7)		5 (8.2)	6 (14.3)	
*Routine/manual*	759 (28.2)	22 (22.0)	33 (25.4)	11 (29.7)	25 (33.1)	12 (26.7)		35 (28.2)		18 (29.5)	8 (19.1)	
*Unemployed*	357 (13.3)	3 (3.0)	15 (11.5)	10 (27.0)	6 (7.5)	14 (31.1)		34 (27.4)		15 (24.6)	11 (26.1)	
*Missing*	316 (11.7)	8 (8.0)	22 (16.9)	6 (16.2)	12 (15.0)	0 (0.0)		18 (14.5)		9 (14.7)	8 (19.1)	
***Smoking***												
*Non-smoker*	1913 (71.1)	95 (95.0)	124 (95.4)	37 (100)	75 (93.8)	37 (82.2)		118 (95.2)		52 (85.3)	28 (66.7)	<0.001
*Smoker*	724 (26.9)	5 (5.0)	4 (3.1)	0 (0.0)	3 (3.7)	8 (17.8)		2 (1.6)		8 (13.1)	14 (33.3)	
*Missing*	54 (2.0)	0 (0.0)	2 (1.5)	0 (0.0)	2 (2.5)	0 (0.0)		4 (3.2)		1 (1.6)	0 (0.0)	
***Inadequate utilization of ANC services***												
*No*	2644 (98.3)	100 (100)	129 (99.2)	36 (97.3)	80 (100)	44 (97.8)		123 (99.2)		61 (100)	42 (100)	0.626
*Yes*	12 (0.5)	0 (0.0)	1 (0.8)	0 (0.0)	0 (0.0)	1 (2.2)		1 (0.8)		0 (0.0)	0 (0.0)	
*Missing*	35 (1.3)	0 (0.0)	0 (0.0)	1 (2.7)	0 (0.0)	0 (0.0)		0 (0.0)		0 (0.0)	0 (0.0)	
**Pregnancy related factors**												
***Current pregnancy problems***												
Anaemia												
*No*	2642 (98.2)	98 (98.0)	128 (98.5)	35 (94.6)	79 (98.8)	45 (100.0)		123 (99.2)		59 (96.7)	41 (97.6)	0.110
*Yes*	15 (0.5)	2 (2.0)	2 (1.5)	1 (2.7)	1 (1.2)	0 (0.0)		1 (0.8)		2 (3.3)	1 (2.4)	
*Missing*	35 (1.3)	0 (0.0)	0 (0.0)	1 (2.7)	1 (0.8)	0 (0.0)		0 (0.0)		0 (0.0)	0 (0.0)	
Diabetes												
*No*	2618 (97.3)	96 (96.0)	127 (97.7)	35 (94.6)	76 (95.0)	44 (97.8)		116 (93.6)		58 (95.1)	38 (90.5)	<0.001
*Yes*	38 (1.4)	4 (4.0)	3 (2.3)	1 (2.7)	4 (5.0)	1 (2.2)		8 (6.4)		3 (4.9)	4 (9.5)	
*Missing*	35 (1.3)	0 (0.0)	0 (0.0)	1 (2.7)	0 (0.0)	0 (0.0)		0 (0.0)		0 (0.0)	0 (0.0)	
Mental health problems												
*No*	2641 (98.1)	100 (100)	130 (100)	36 (97.3)	80 (100)	45 (100)		124 (100)		61 (100)	42 (100)	0.746
*Yes*	15 (0.6)	0 (0.0)	0 (0.0)	0 (0.0)	0 (0.0)	0 (0.0)		0 (0.0)		0 (0.0)	0 (0.0)	
*Missing*	35 (1.3)	0 (0.0)	0 (0.0)	1 (2.7)	0 (0.0)	0 (0.0)		0 (0.0)		0 (0.0)	0 (0.0)	
***Parity***												
*Primiparous*	1187 (44.1)	50 (50.0)	31 (23.9)	10 (27.0)	34 (42.5)	21 (46.7)		47 (37.9)		25 (41.0)	22 (52.4)	<0.001
*1–3*	1399 (52.0)	48 (48.0)	93 (71.5)	26 (70.3)	46 (57.5)	22 (48.9)		63 (50.8)		35 (57.4)	19 (45.2)	
*>3*	85 (3.2)	2 (2.0)	6 (4.6)	1 (2.7)	0 (0.0)	2 (4.4)		14 (11.3)		1 (1.6)	1 (2.4)	
*Missing*	20 (0.7)	0 (0.0)	0 (0.0)	0 (0.0)	0 (0.0)	0 (0.0)		0 (0.0)		0 (0.0)	0 (0.0)	
***Previous pregnancy problems***												
*No*	2111 (78.5)	84 (84.0)	91 (70.0)	28 (75.7)	67 (83.8)	36 (80.0)		94 (75.8)		52 (85.3)	31 (73.8)	0.585
*Yes*	443 (16.5)	13 (13.0)	30 (23.1)	7 (18.9)	10 (12.5)	5 (11.1)		24 (19.4)		7 (11.5)	9 (21.4)	
*Missing*	137 (5.1)	3 (3.0)	9 (6.9)	2 (5.4)	3 (3.7)	4 (8.9)		6 (4.8)		2 (3.3)	2 (4.8)	
***Pre-existing medical problems***												
*No*	2087 (77.6)	84 (84.0)	109 (83.9)	32 (86.5)	60 (75.0)	40 (88.9)		96 (77.4)		50 (82.0)	33 (78.6)	0.513
*Yes*	577 (21.4)	16 (16.0)	20 (15.4)	4 (10.8)	20 (25.0)	5 (11.1)		27 (21.8)		11 (18.0)	9 (21.4)	
*Missing*	27 (1.0)	0 (0.0)	1 (0.8)	1 (2.7)	0 (0.0)	0 (0.0)		1 (0.8)		0 (0.0)	0 (0.0)	

Total sample = 3310 (controls);

f P-value of the Pearson's chi square test; SD – Standard Deviation;

† Body mass index at the time of booking; ANC – Antenatal care; the non-white (non-Asian) group included women from any other black background and other ethnic groups such as Hispanics/Latin Americans.

Unadjusted univariable analysis showed that women belonging to black African ethnic minority group had twice the odds of severe maternal morbidity compared with the white European women (Table 3). Compared to the white European women, the odds of severe maternal morbidity were 79% higher among the black Caribbeans, 73% higher among Bangladeshi women, 59% higher among the other non-white (non-Asian) group (includes women from any other black background and other ethnic groups such as Hispanics/Latin Americans) and 37% higher among the Pakistani women.

**Table 3:** Unadjusted odds ratios for severe maternal morbidity.

Risk factors	No (%) Cases	No (%) Controls	Unadjusted odds ratio OR (95% CI)
**Ethnicity**			
White European	1303 (74.3)	2691 (81.3)	1
Indian	47 (2.7)	100 (3.0)	0.97 (0.68 to 1.38)
Pakistani	86 (4.9)	130 (3.9)	1.37 (1.03 to 1.81)
Bangladeshi	31 (1.8)	37 (1.1)	1.73 (1.07 to 2.80)
Other Asian	50 (2.9)	80 (2.4)	1.29 (0.90 to 1.85)
Black Caribbean	39 (2.2)	45 (1.4)	1.79 (1.16 to 2.76)
Black African	123 (7.0)	124 (3.8)	2.05 (1.58 to 2.65)
Other non - white (not Asian)	47 (2.7)	61 (1.8)	1.59 (1.08 to 2.34)
Mixed	27 (1.5)	42 (1.3)	1.33 (0.82 to 2.16)
**Pregnancy related factors**			
***Current pregnancy problems***			
Anaemia			
*No*	1716 (97.9)	3250 (98.2)	1
*Yes*	24 (1.4)	24 (0.7)	1.89 (1.07 to 3.35)
*Missing*	13 (0.7)	36 (1.1)	0.68 (0.36 to 1.29)
Diabetes			
*No*	1685 (96.1)	3208 (96.9)	1
*Yes*	55 (3.1)	66 (2.0)	1.59 (1.10 to 2.28)
*Missing*	13 (0.7)	36 (1.1)	0.69 (0.36 to 1.30)
Mental health problems			
*No*	1731 (98.8)	3259 (98.4)	1
*Yes*	9 (0.5)	15 (0.5)	1.13 (0.49 to 2.59)
*Missing*	13 (0.7)	36 (1.1)	0.68 (0.36 to 1.29)
***Parity***			
*Primiparous*	717 (40.9)	1427(43.1)	0.96 (0.86 to 1.09)
*1 to 3*	912 (52.0)	1751 (52.9)	1
*>3*	121 (6.9)	112(3.4)	2.07 (1.58 to 2.71)
*Missing*	3 (0.2)	20 (0.6)	0.29 (0.09 to 0.97)
***Previous pregnancy problems***			
*No*	1338 (76.3)	2594 (78.3)	1
*Yes*	406 (23.2)	548 (16.6)	1.44 (1.24 to 1.66)
*Missing*	9 (0.5)	168 (5.1)	0.10 (0.05 to 0.20)
***Pre-existing medical problems***			
*No*	1238 (70.6)	2591 (78.3)	1
*Yes*	509 (29.1)	689 (20.8)	1.55 (1.35 to 1.77)
*Missing*	6 (0.3)	30 (0.9)	0.42 (0.17 to 1.01)
**Behavioural factors, demographic factors and BMI**			
*Smoking status*			
*Non-smoker*	1364 (77.8)	2479 (74.9)	1
*Smoker*	361 (20.6)	768 (23.2)	0.85 (0.74 to 0.98)
*Missing*	28 (1.6)	63 (1.9)	0.81 (0.52 to 1.27)
***Inadequate utilization of ANC***			
*No*	1722 (98.3)	3259 (98.4)	1
*Yes*	18 (1.0)	15 (0.5)	2.27 (1.14 to 4.52)
*Missing*	13 (0.7)	36 (1.1)	0.68 (0.36 to 1.29)
***Socioeconomic status (occupational classification (NS-SEC))***			
*Managerial*	450 (25.7)	844 (25.5)	1
*Intermediate*	357 (20.3)	679 (20.5)	0.99 (0.83 to 1.17)
*Routine and manual*	444 (25.3)	923 (27.9)	0.90 (0.77 to 1.06)
*Unemployed*	252 (14.4)	465 (14.1)	1.02 (0.84 to 1.23)
*Missing*	250 (14.3)	399 (12.0)	1.18 (0.97 to 1.43)
***Age***			
*<20 years*	103 (5.9)	156 (4.7)	1.45 (1.12 to 1.88)
*20 – 34 years*	1106 (63.1)	2436 (73.6)	1
*≥ 35 years*	544 (31.0)	698 (22.1)	1.72 (1.50 to 1.96)
*Missing*	0 (0.0)	20 (0.6)	Omitted
***BMI*** †			
*<18.5*	49 (2.8)	92 (2.8)	1.04 (0.73 to 1.48)
*18.5 – 30*	1214 (69.2)	2373 (71.7)	1
*≥ 30*	348 (19.9)	597 (18.0)	1.14 (0.98 to 1.32)
*Missing*	142 (8.1)	248 (7.5)	1.12 (0.90 to 1.39)

Total sample  = 5063;

† Body mass index at the time of booking (kg/m^2^); ANC – Antenatal care; OR – odds ratio; CI – Confidence Interval; 1 denotes the baseline comparison group; the non-white (non-Asian) group included women from any other black background and other ethnic groups such as Hispanics/Latin Americans.

Having accounted for other factors and possible confounders sequentially, the fully adjusted model 4 shows that compared with white European women, the odds of severe maternal morbidity were 83% higher among the black African women, 80% higher among black Caribbean, 74% higher in Bangladeshi, and 56% and 43% higher in the other non-white (non-Asian) and Pakistani groups, respectively (Table 4). There was remarkably little change in odds ratios with adjustment for other factors across the models suggesting that other socio-demographic and clinical factors had little or no confounding effects.

**Table 4:** Adjusted odds ratios for severe maternal morbidity by ethnic group.

Risk factors	Unadjusted	Model-1	Model-2	Model-3	Model-4
	OR (95% CI)	aOR (95% CI)	aOR (95% CI)	aOR (95% CI)	aOR (95% CI)
**Ethnicity**					
White European	1	1	1	1	1
Indian	0.97 (0.68 to 1.38)	0.98 (0.69 to 1.40)	0.95 (0.66 to 1.36)	0.94 (0.66 to 1.35)	1.00 (0.70 to 1.44)
Pakistani	1.37 (1.03 to 1.81)	1.38 (1.04 to 1.84)	1.33 (0.99 to 1.77)	1.32 (0.98 to 1.76)	1.43 (1.07 to 1.92)
Bangladeshi	1.73 (1.07 to 2.80)	1.67(1.02 to 2.74)	1.61 (0.98 to 2.65)	1.65 (1.00 to 2.72)	1.74 (1.05 to 2.88)
Other Asian	1.29 (0.90 to 1.85)	1.32 (0.92 to 1.90)	1.27 (0.88 to 1.83)	1.28 (0.89 to 1.84)	1.27 (0.87 to 1.84)
Black Caribbean	1.79 (1.16 to 2.76)	1.89 (1.21 to 2.94)	1.84 (1.18 to 2.88)	1.90 (1.21 to 2.97)	1.80 (1.14 to 2.82)
Black African	2.05 (1.58 to 2.65)	1.90 (1.46 to 2.48)	1.80 (1.38 to 2.35)	1.83 (1.39 to 2.39)	1.83 (1.39 to 2.40)
Other non - white (non-Asian)	1.59 (1.08 to 2.34)	1.61 (1.09 to 2.38)	1.56 (1.05 to 2.31)	1.55 (1.04 to 2.30)	1.56 (1.05 to 2.33)
Mixed	1.33 (0.82 to 2.16)	1.35 (0.82 to 2.23)	1.37 (0.83 to 2.26)	1.35 (0.82 to 2.23)	1.28 (0.77 to 2.13)
**Pregnancy related factors**					
***Current pregnancy problems***					
Anaemia					
*No*		1	1	1	1
*Yes*		1.80 (0.99 to 3.26)	1.82 (1.01 to 3.31)	1.83 (1.01 to 3.32)	1.82 (1.00 to 3.32)
Diabetes					
*No*		1	1	1	1
*Yes*		1.26 (0.87 to 1.83)	1.26 (0.87 to 1.83)	1.27 (0.87 to 1.85)	1.20 (0.82 to 1.75)
***Previous pregnancy problems***					
*No*		1	1	1	1
*Yes*		1.28 (1.09 to 1.50)	1.28 (1.09 to 1.50)	1.28 (1.09 to 1.50)	1.27 (1.08 to 1.50)
***Pre-existing medical problems***					
*No*		1	1	1	1
*Yes*		1.53 (1.33 to 1.75)	1.55 (1.35 to 1.77)	1.55 (1.35 to 1.77)	1.54 (1.34 to 1.77)
*Parity*					
*Primiparous*		0.98 (0.86 to 1.12)	0.98 (0.85 to 1.11)	0.97 (0.85 to 1.11)	1.00 (0.87 to 1.15)
*1–3*		1	1	1	1
*>3*		1.79 (1.35 to 2.37)	1.83 (1.38 to 2.42)	1.82 (1.37 to 2.41)	1.64 (1.23 to 2.20)
**Behavioural factors, demographic factors and BMI**					
*Smoking*					
*Non-smoker*			1	1	1
*Smoker*			0.84 (0.73 to 0.98)	0.86 (0.74 to 1.01)	0.88 (0.75 to 1.03)
***Inadequate utilization of ANC services***					
*No*			1	1	1
*Yes*			2.07 (1.02 to 4.23)	2.04 (1.00 to 4.17)	1.97 (0.96 to 4.04)
***Socioeconomic status (occupational classification (NS-SEC))***					
*Managerial*				1	1
*Intermediate*				0.99 (0.83 to 1.18)	1.04 (0.87 to 1.24)
*Routine and manual*				0.88 (0.75 to 1.04)	0.94 (0.79 to 1.11)
*Unemployed*				0.91 (0.74 to 1.12)	0.92 (0.74 to 1.15)
***Age***					
*<20 years*					1.63 (1.23 to 2.17)
*20 – 34 years*					1
*≥ 35 years*					1.58 (1.37 to 1.82)
***BMI*** †					
*<18.5*					1.11 (0.77 to 1.60)
*18.5 – 30*					1
*≥ 30*					1.03 (0.88 to 1.21)

† Body mass index at the time of booking OR  =  odds ratio aOR  =  adjusted odds ratio CI  =  Confidence Interval ANC  =  Antenatal Care 1 denotes the baseline comparison group the non-white (non-Asian) group included women from any other black background and other ethnic groups such as Hispanics/Latin Americans.

Model-1: adjusted for anaemia in current pregnancy, diabetes in current pregnancy, previous pregnancy problems, pre-existing medical problems and parity.

Model-2: adjusted for all variables included in model-1, smoking and inadequate utilization of ANC services.

Model-3: adjusted for variables included in models 1 and 2 plus socioeconomic status.

Model-4: all variables included in models 1, 2 and 3 plus age and BMI.

Anaemia in the current pregnancy, previous pregnancy problems, pre-existing medical conditions, parity >3 and being younger and older were independent risk factors for severe maternal morbidity. However, the odds of severe morbidity did not differ according to the socioeconomic status of the women, between smokers and non-smokers or by BMI.

Women with inadequate utilisation of ANC services were twice as likely to be at risk of severe morbidity compared to women who adequately utilized the services (adjusted OR = 1.97; 95% CI = 0.96 to 4.04) although this result was not statistically significant. The odds of inadequate utilization of ANC services was higher among black African (OR = 4.46; 95% CI = 1.47 to 11.48) and Caribbean (OR = 4.35; 95% CI = 0.49 to 18.19) women compared with white European women.

## Discussion

This study which included 1,753 women with severe maternal morbidity reported through the nationwide surveillance system, UKOSS, demonstrated a significantly higher risk of severe morbidity between 43% to 83% among women belonging to the Pakistani, Bangladeshi, black Caribbean, black African and other non-white (non-Asian) ethnic minority groups compared with white European women. Although not statistically significant, the risk was 28% higher in the mixed group and 27% higher in the other Asian group. However, the risk among Indian women was the same as that of white European women. Factors including inadequate utilisation of ANC, parity, smoking, pre-existing medical problems and age explained a small part of this increased risk, but socioeconomic status did not influence the association. In addition to ethnic background, anaemia during pregnancy, pre-existing medical problems, previous pregnancy problems, high parity, and younger and older age were all independent risk factors for severe maternal morbidity.

The findings of this study are similar to those of the earlier UKOSS study in which Knight et al. demonstrated the unadjusted relative risk of having a ‘near-miss’ condition to be more than two times higher in black Caribbean and African women and about 49% higher among Pakistani women than the white Europeans [1]. The ethnic disparities in severe maternal morbidity observed in this study are also supported by studies conducted in other parts of the world. Studies in the USA have consistently demonstrated a higher risk of severe maternal morbidity (almost 40% higher) among African-American women compared to the white population [5], [26]–[30]. A study conducted in Germany (2001–2007) among 441,199 mothers demonstrated a higher risk of severe morbidity (sepsis, eclampsia, hysterectomy and haemorrhage) among migrant women from Asia, Africa/Latin America and the Middle East compared to German women [31]. A nationwide prospective study from the Netherlands demonstrated a 1.3 fold higher risk of severe maternal morbidity among the ‘non-Western immigrant population’ (defined as all immigrants other than those from the European countries, North America, Japan and Indonesia) compared to Western women [32]. The risk was found to be markedly higher among women from sub-Saharan Africa [32]. This increased risk was partly explained by the socio-demographic, lifestyle and immigration related factors [32], a qualitative study attributed the risk to low health literacy, language barriers, non-familiarity with the health system [33], and substandard care was found to be a risk factor during audits [34].

Analysis of maternal mortality in the UK (2003–2008) showed that the likelihood of dying following an episode of severe morbidity was higher among black Caribbean and African women compared with white European women [35]. While the exact cause of this increased risk is not known, the authors suggested a possible role of inadequate access/utilisation of healthcare services among the black ethnic groups [35]. In this study, we were able to demonstrate quantitatively that inadequate utilisation of ANC services is an independent risk factor for severe maternal morbidity and may explain some of the observed increased risk among the ethnic minority groups. This finding is supported by the results of two cross-sectional studies in England. Cresswell et al. using electronic patient record data from East London, showed that the odds of late booking (after 12 weeks gestation) was higher among women from the ethnic minority groups compared to British white women even among the black Caribbean/African women who were born in the UK and spoke English [36]. Rowe et al. identified women from 198 hospitals across England and demonstrated a higher rate of late booking among women from black Caribbean and African ethnic backgrounds compared to white women [37]. The small number of pregnant women with concealed pregnancy/minimal ANC/late booking problems in our dataset precludes a definitive finding, but suggests a hypothesis that could be tested in further studies.

We found other factors that also independently increased the risk of severe morbidity. Women who had anaemia during pregnancy and those with previous pregnancy or medical problems were at a higher risk of experiencing a severe morbidity condition than women who did not. A number of studies both in the UK and abroad have demonstrated associations between anaemia during pregnancy and severe morbidity. A case-control study in Scotland showed that after controlling for other risk factors, women with anaemia during pregnancy (most likely iron deficiency anaemia) were at a higher risk of severe sepsis [38]. Similarly, a national study in Norway using data from the population based registry, showed a significant association between anaemia during pregnancy (haemoglobin <9 g/dl) and severe obstetric haemorrhage [39]. A further case-control study conducted in South East England to identify the predictors of severe maternal morbidity found that women who were taking iron supplements at booking were five times more likely to develop a ‘near-miss’ condition (severe pre-eclampsia and peripartum haemorrhage) compared with women who did not [40].

A study in the USA using National Hospital Discharge Survey data for women giving birth between 1993 and 1997 [41] and a case-control study in 19 maternity units in England (1997–98) [40] showed that women who had experienced complications during one or more previous pregnancies were at a higher risk of experiencing subsequent severe morbidity. Studies in the UK and USA have reported a significantly higher risk of developing a ‘near-miss’ condition among women with pre-existing medical conditions (hypertension, diabetes, extrinsic asthma, malignancy) [30], [40], [42]. While these are independent risk factors importantly, they did not explain the observed ethnic differences in severe maternal morbidity in the UK.

As the population of ethnic minority groups in the UK continues to increase, it is important to focus on these ethnic disparities in severe maternal morbidity. The observed disparities could be due to inadequate utilization of ANC services, but the known risk factors for severe maternal morbidity explained very little of this disparity. There could be residual confounding in our study due to factors that were not measured well (for example socioeconomic status, inadequate utilization of ANC) or not measured at all (for example education level of the women, cultural factors and social status of women). The variable ‘inadequate utilisation of ANC’ included women who did not seek any antenatal care, but we did not have information about women who were irregular attendees or defaulters in care seeking. Further, it is important to understand other elements of the care pathway to disentangle the reasons for inadequate utilisation of ANC which could be lack of information, language barriers or cultural differences. Studies globally have associated inadequate provision and utilization of healthcare services [43]–[45], certain cultural practices [44]–[46], lower level of education [43], [44] and low social status of women [44], [45] with increased risk of maternal morbidity and mortality. Thus, it is important to understand the role of these factors in increasing the risk of severe morbidity among women belonging to BME groups in the UK.

Policies have been developed by the National Institute for Health and Care Excellence (NICE) for cardiovascular disease (CVD) screening and prevention in which NICE recommends that certain ethnic groups that are at a higher risk of CVD should be targeted for primary prevention and are considering measures to develop a UK population based risk scoring system taking into account the ethnic differences in CVD prevalence [47]. It may be time to consider the same for care in pregnancy. In addition to providing a quantitative estimation of the risk of severe morbidity in the different minority ethnic groups in the UK, this study also elucidates the independent contribution of preventable factors such as inadequate utilisation of ANC services and anaemia during pregnancy to increased maternal morbidity.

### Strengths and limitations

In this national study, the approach used to identify severe maternal morbidity as an aggregate of ten conditions directly related to maternal mortality in the UK addresses the challenges related to the difficulty of defining ‘severe maternal morbidity’. However, potential limitations due to non-inclusion of the indirect causes of maternal mortality in the UK such as cardiac disease, psychiatric illness and suicide [48] remain. As discussed above, residual confounding due to factors not included or adequately measured remains a possibility.

## Conclusion

In summary, this national study clearly demonstrates an increased risk of severe maternal morbidity among women of all ethnic minority backgrounds in the UK except among women of Indian and mixed origins, and provides important insights into the independent association of inadequate utilisation of ANC, high parity and pregnancy in younger and older age with the odds of severe maternal morbidity. This provides a focus for further research into possible pathways of prevention of severe maternal morbidity.
